# P01-008 – FMF genotype-phenotype correlations in Germany

**DOI:** 10.1186/1546-0096-11-S1-A12

**Published:** 2013-11-08

**Authors:** M Jeske, P Lohse, T Kallinich, T Berger, C Rietschel, D Holzinger, C Kamlah, P Lankisch, R Berendes, G Dückers, G Horneff, E Lilienthal, JP Haas, A Giese, F Dressler, J Berrang, C Pütter, L Braunewell, U Neudorf, T Niehues, E Lainka

**Affiliations:** 1 Children's Hospital, University Duisburg-Essen, Essen, Germany; 2 Labor Blessing und Partner, Singen, Germany; 3 Charité Berlin, Children's hospital, Berlin, Germany; 4 Children's Hospital, University Witten/Herdecke, Datteln, Germany; 5 Department of Pediatric Rheumatology, Clementine Hospital , Frankfurt, Germany; 6 Children's Hospital, University Münster, Münster, Germany; 7 Children's Hospital, UKE Hamburg, Hamburg, Germany; 8 Pediatric Rheumatology, University Düsseldorf, Düsseldorf, Germany; 9 Pediatric Rheumatology, St. Marien's Children's Hospital, Landshut, Germany; 10 Pediatric Immunology and Rheumatology, HELIOS Children's hospital, Krefeld, Germany; 11 Centre of Pediatric Rheumatology, Asklepios Clinic, St. Augustin, Germany; 12 Children's Hospital, Ruhr-University , Bochum, Germany; 13 German Center for Pediatric Rheumatology, Children's Hospital, Garmisch-Partenkirchen, Germany; 14 Marienhospital Herne, Ruhr-University Bochum, Herne, Germany; 15 Pediatric Rheumatology Clinic, Hannover Medical School, Hannover, Germany; 16 Pediatric Rheumatology, Westfaelisches Kinderzentrum , Dortmund, Germany; 17 Institute for Medical Informatics, Biometry and Epidemiology, University Hospital, Essen, Germany

## Introduction

Familial Mediterranean fever (FMF) is one the most common autoinflammatory disease (AID). Pathogenomic relevant mutations in the MEFV gene show autosomal recessive inheritance, but co-dominant mutations have been described.

## Objectives

We aimed to evaluate correlations between ethnic origin, phenotype and genotype for FMF patients in the German AID-Net-registry.

## Methods

We used two common scoring systems modified for children (Mor et al., Pras et al.) to assess disease severity in 243 FMF patients of the AID-Net-registry. For the four most frequent mutations, we tested for a correlation of the genotype with the phenotype, mean CRP and ethnic origin, respectively. Furthermore, we evaluated the applicability of the two severity scores for children.

## Results

Among the 243 patients, we detected a total of 433 pyrin mutations and 22 different sequence variants, including one new mutation (p.Gly488Asp). The four most frequent alterations were p.Met694Val (55%, n=238), p.Met680lle (12%, n=52), p.Val726Ala (10%, n=44) and p.Glu148Gln (8%, n=34). Ethnic origin could be determined in 224 cases; the prevailing ancestry was Turkish (83%, n=185), 8% (n=18) were Lebanese. P.Met694Val in homozygous form (n=74; 30.5%) was correlated with a more severe disease activity, based on the score by Mor, as well as with a higher mean CRP (74 mg/l, n=60, 31 mg/l, n=59) compared to patients without this mutation (p=0.01 and p<0.01, respectively). The score suggested by Pras did not yield a significant genotype-phenotype correlation; indeed, the two scoring systems were inconsistent with each other (κ<0.07). Although a typical distribution of mutations in different ethnic populations was obvious, this trend was not statistically significant, probably due to the divergent number of cases.

## Conclusion

The homozygous p.Met694Val substitution was associated with a more severe disease activity. There was no origin-genotype correlation in this FMF population. The well-known severity scores for children (Mor, Pras) are inconsistent.

## Disclosure of interest

M. Jeske: None Declared, P. Lohse: None Declared, T. Kallinich: None Declared, T. Berger: None Declared, C. Rietschel: None Declared, D. Holzinger : None Declared, C. Kamlah: None Declared, P. Lankisch: None Declared, R. Berendes: None Declared, G. Dückers: None Declared, G. Horneff Consultant for: financial support for clinical trials from Abbott, Pfizer and Roche, E. Lilienthal: None Declared, J. Haas Consultant for: Pfizer, Roche and Novartis, A. Giese: None Declared, F. Dressler: None Declared, J. Berrang: None Declared, C. Pütter: None Declared, L. Braunewell: None Declared, U. Neudorf: None Declared, T. Niehues: None Declared, E. Lainka: None Declared

